# Supplementary material for: Ultrafast dynamic stark shift of an exciton-polariton condensate
Source: Nat Commun. 2026 Jan 28;17:2089. doi: 10.1038/s41467-026-68703-x (PMC12953906; doi:10.1038/s41467-026-68703-x)
Supplement: Supplementary file 1 — Supplementary Information [file 41467_2026_68703_MOESM1_ESM.pdf]

Supplementary information:

Ultrafast Dynamic Stark Shift of an Exciton-Polariton Condensate

Sarit Feldman[1], Dmitry Panna[1], Nadav Landau[1], Sebastian Brodbeck[2], Sebastian Klembt[2], Christian Schneider[2,3], Sven Höfling[2] and Alex Hayat[1]

1. Department of Electrical and Computer Engineering, Technion – Israel Institute of Technology, Haifa, 3200003, Israel

2. Technische Physik, Physikalisches Institut and Wilhelm Conrad Röntgen Research Center for Complex Material Systems, Universität Würzburg, D-97074 Würzburg, Germany

3. Institute of Physics, Carl von Ossietzky University, 26129 Oldenburg, Germany

Supplementary Note 1: Calculation of differential reflectivity spectra based on the theory of coherent transients

The theory of coherent transients provides a semiclassical description of the polarization in ultrafast pump-probe schemes[1,2,3]. The model predicts the formation of spectro-temporal oscillations in the differential absorption or transmission with a spectral period trending, roughly, inversely to the pump-probe delay at times $\Delta t < 0$, when the pump precedes the probe on the sample. Such oscillations in the spectral vicinity of excitonic and polaritonic resonances have been demonstrated in various pump-probe measurements[2,4,5,6,7]. The characteristics of the oscillation fringes, manifested in their absolute amplitude and in the decay in their visibility with increasing pump delay and with spectral detuning, are dictated by both the carrier lifetime and polarization coherence time.

In this model, the susceptibility $\chi(\omega)$ is dictated by the pump while the weaker probe is treated perturbatively, yielding the absorption spectra $\alpha(\omega) = \mathrm{Im}\dfrac{\Omega_{\mathrm{p}}}{2\varepsilon_0 c} < \chi(\omega) >$ where Ω_{p} is the pump frequency. $< \chi(\omega) >$ is characterized by the dipole coupling to the pump, the polariton linewidth and the energy detuning between the pump and the polariton branch. Our calculation of $\alpha(\omega)$ follows the model, while we represent the absorption in the absence of a pump as a Lorentzian of linewidth γ: $\alpha_0(\omega) = \dfrac{\Omega_{\mathrm{p}}}{2\varepsilon_0 c}\mathrm{Im}\left\{\dfrac{i\mu^2}{\gamma + i(E_{\mathrm{pol}}/\hbar - \omega)}\right\}$

where μ is the polariton dipole moment and E_{pol} is the polariton energy. γ was found from the differential reflectivity spectrum at short pump-probe delay $\Delta t \sim 0$.

We derive the differential reflectivity spectrum from the calculated absorption spectra $\alpha(\omega)$, $\alpha_0(\omega)$ according to $\Delta R / R = \dfrac{\alpha_0 - \alpha}{1-\alpha_0}$. In the simulation of $\Delta R / R$ at varying probe intensity, only the linewidth and the energy of the reflectance dip were allowed as free fitting parameter.

Supplementary Note 2: Two-photon absorption-induced spectral feature at positive time delays

Two-photon absorption (TPA) of the pump is expected to be negligible in the QWs. Based on TPA coefficients reported by Nathan et al[8], we estimate that while most of the pump power is absorbed in the top distributed Bragg reflector (DBR), only ~0.1% is absorbed by the quantum wells. This estimate is corroborated by photoluminescence (PL) measurements under pump-only excitation in our setup. As shown in Supplementary Figure 1, the PL spectrum exhibits a distinct feature at 1729 meV, close to the bandgap of the $Al_{0.2}Ga_{0.8}As$ DBRs, while no signal is detected at the lower polariton (LP) and upper polariton (UP) energies of 1605.3 meV and 1618.9 meV, respectively.

TPA-induced carriers in the DBR account for the deviation of the measured PL at 1729 meV from the nominal cryogenic band gap of $Al_{0.2}Ga_{0.8}As$ (1768 meV); according to Bennett et al.[9], a carrier density sufficient to induce a bandgap shrinkage of ~30 meV corresponds to a ~15% modification of the DBR refractive index, leading to an expected red shift of 1–2 meV in the polariton energy. This shift is consistent with the feature observed at positive delays in Fig. 3c,d in the main text and in Supplementary Figure 4c,d.

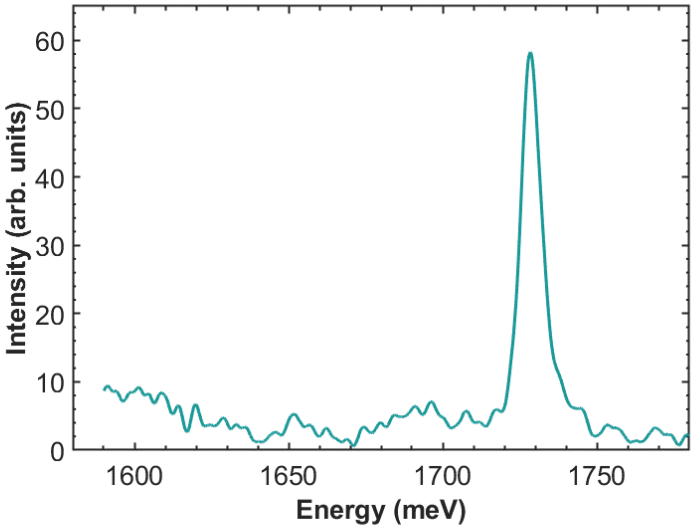

Supplementary Figure 1 | PL spectrum excited with 1550 meV pump. Pump-excited PL was collected from the sample using our experimental setup. A peak corresponding to the bandgap of $Al_{0.2}Ga_{0.8}As$, altered by carrier-induced shrinkage, indicates TPA of the pump in the DBR. No PL is detected for the LP and UP modes at 1605.3 meV and 1618.9 meV, respectively.

Supplementary Note 3: LP energy versus excitation power in PL measurements

The LP energy versus excitation power obtained in PL measurements of our sample at δ≈0 is shown in Supplementary Figure 2. The results were obtained using a resonant pump incident on the sample at 45°, tuned to 1653 meV to match the cavity dip associated with the 45° stopband, and the PL was collected using an objective lens. We note that the discontinuity in the blueshift observed here as well as in PL measurements reported for other samples[10], is an apparent effect due to pulsed excitation; in fact, the energy of the LP varies smoothly with excitation power. This effect results from temporal integration of the measured PL, whereas in our differential reflectivity measurements the nature of the effect is spatial, as described in the main text.

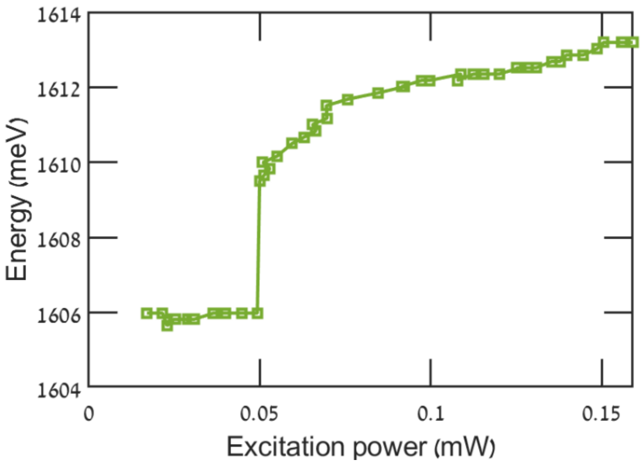

Supplementary Figure 2 | LP energy versus average excitation power in direct PL measurements. The results are shown for cavity-exciton detuning $\delta\approx0$. The excitation beam, impinging on the sample at oblique incidence, was centered at 1653 meV, corresponding to the cavity dip within the 45° stopband. The resulting PL was collected with an objective lens. A sharp blueshift is observed, as is the case in our experiment for δ=5.1 meV.

Supplementary Note 4: Calculation of AC Stark shift in the LP and UP

To estimate the Stark shift as a function of pump intensity, a reference spectral profile, corresponding to the LP or UP reflectivity dip without the Stark beam was subtracted from a profile shifted by varying Stark shifts, $\Delta E_{\mathrm{Stark}}$. The estimated shift $\Delta E_{\mathrm{Stark}}$ was found according to the result which best fitted the measured differential reflectivity. The results were compared to the theoretical dependence of the Stark shift on pump intensity, indicated by dashed lines in Fig. 5 in the main text. The shift was derived from the eigenstates of the following Hamiltonian representing the system of an exciton interacting with the microcavity vacuum field and with the pump, with the pump coupling efficiency as a fitting parameter to the calculated results:

$$H = \begin{pmatrix} E_{\mathrm{x}} & \hbar\Omega_{\mathrm{R}} & \hbar\Omega_{\mathrm{p}} \\ \hbar\Omega_{\mathrm{R}} & E_{\mathrm{c}} & 0 \\ \hbar\Omega_{\mathrm{p}} & 0 & E_{\mathrm{p}} \end{pmatrix} \tag{1}$$

E_{x}, E_{C} and E_{p} are the exciton energy, cavity mode and energy of the Stark beam, respectively. Ω_R represents the dipole interaction strength of the exciton with the cavity photon, and Ω_{p} is the interaction strength with the Stark field. The pump-exciton coupling coefficient was set as a free parameter. The calculated theoretical Stark shift is given by the difference between the eigenvalues of H, $E_{LP,S}$ and $E_{UP,S}$, and the energy of the unperturbed LP and UP energy, E_{LP} and E_{UP}:

$$\begin{aligned} \Delta E_{LP} &= E_{LP,S} - E_{LP} \\ \Delta E_{UP} &= E_{UP,S} - E_{UP} \end{aligned} \tag{2}$$

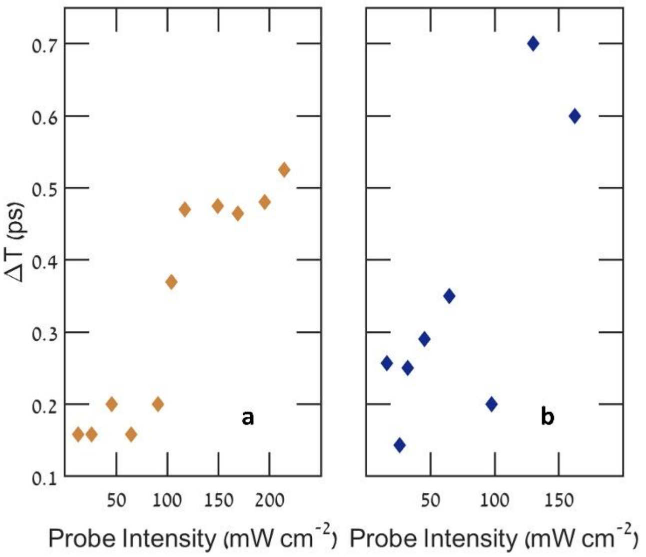

Supplementary Figure 3 | Pump-probe delay at which the Stark effect on the LP is maximal (a) at cavity-exciton detuning δ=+5.1 meV **(b)** at cavity-exciton detuning δ~0. The delay increases above condensation threshold, when the effect is manifested in PL from the condensate. The delay times above condensation threshold are slightly shorter for the exciton-like detuning of δ = +5.1 meV than for $\delta \approx 0$.

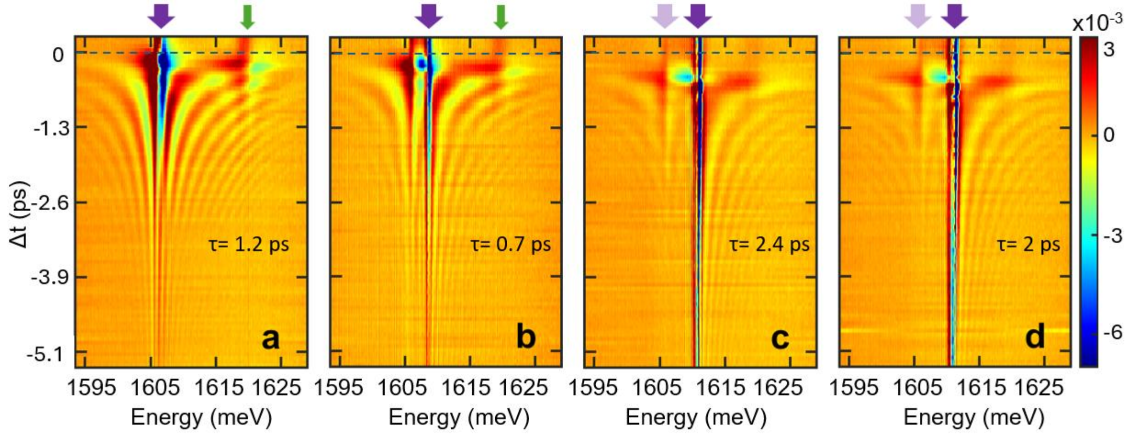

Supplementary Figure 4 | Measured normalized differential reflectivity at cavity-exciton detuning δ~0. Results in a-d are shown for an average probe intensity of 16 mW cm^{-2}, 45 mW cm^{-2}, 129 mW cm^{-2} and 162 mW cm^{-2}, respectively and for a pump peak intensity of 10.2 GW cm^{-2}. The LP and the UP are represented by thick purple arrows and green narrow arrows, respectively. The bright thick arrows in (c,d) represent the LPs in the uncondensed state. The dashed lines mark pump-probe temporal overlap, Δt=0. τ represents the decay time constant of the oscillations.

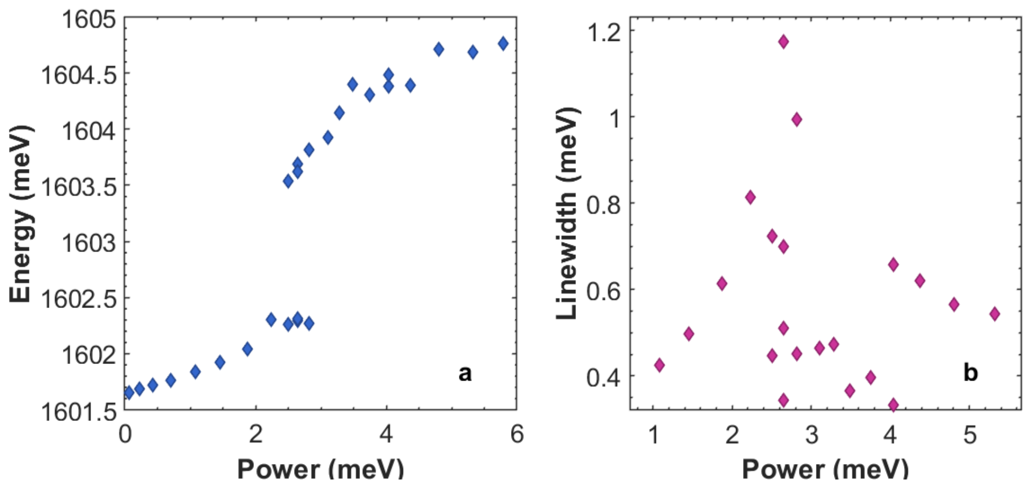

Supplementary Figure 5 | Energy and linewidth of PL versus average excitation power. The energy **(a)** and linewidth **(b)** were extracted from angle-resolved PL spectral measurements of our sample at cavity-exciton detuning δ=−2.9 meV with off-resonant pulsed excitation. The onset of condensation is accompanied by a discontinuity in the blueshift and linewidth narrowing.

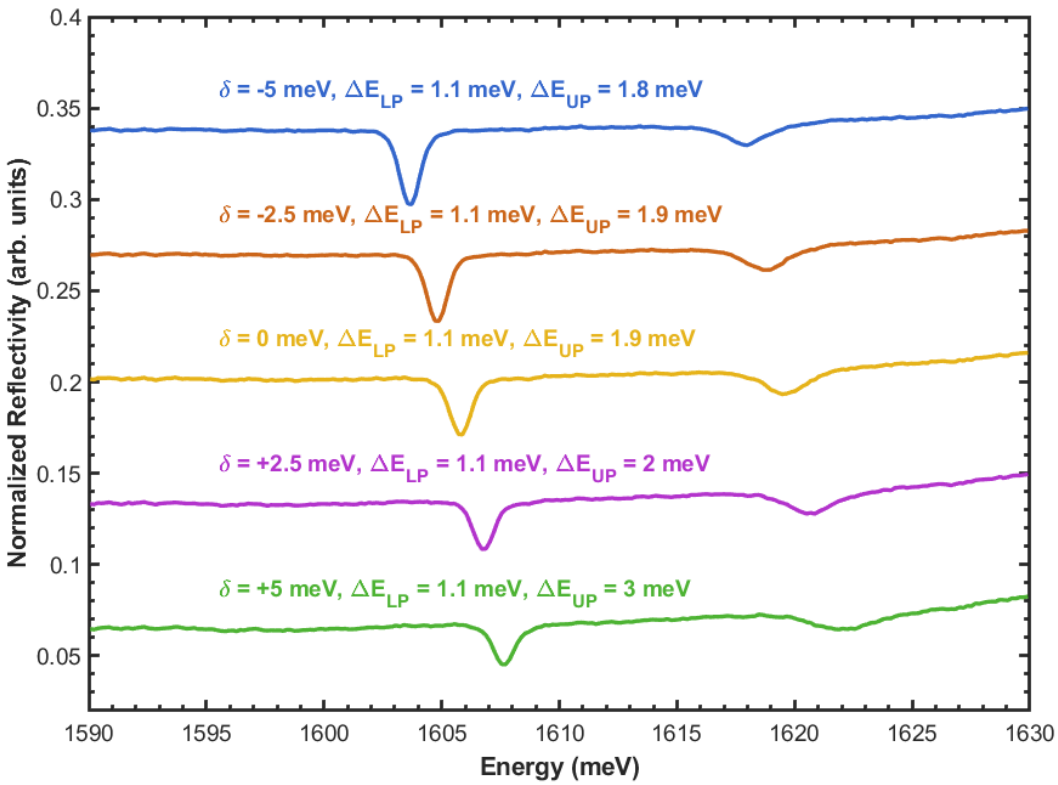

Supplementary Figure 6 | Normalized reflectivity spectra at varying detuning. The profiles display the measured reflectivity spectra acquired at T=4K for different exciton-cavity detuning values with a broadband light source. The labels δ, ΔE_{LP} and ΔE_{UP} denote the respective values of the detuning, the extracted linewidth of the LP and the extracted linewidth of the UP.

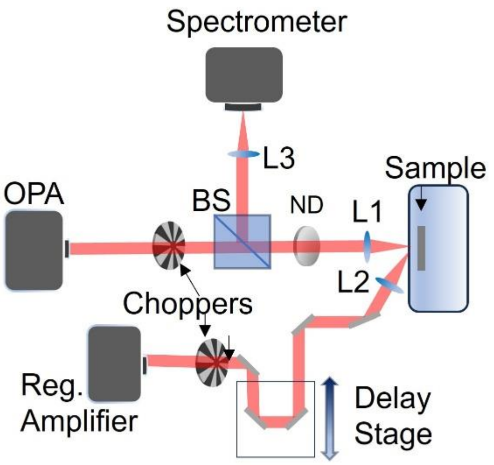

Supplementary Figure 7 | Experimental sketch of the pump-probe differential spectroscopy measurements setup. The OPA, pumped by the regenerative amplifier, delivers the probe beam, which is focused on the sample by lens L1 and reflected towards the spectrometer. The pump inducing the Stark shift, delivered by the regenerative amplifier, impinges on the sample at oblique incidence and is focused by lens L2. Optical choppers control the alternation between pump and probe.

Supplementary References

[1] Fluegel, B., et al. Femtosecond studies of coherent transients in semiconductors. *Physical review letters* **59**, 2588 (1987).

[2] Lindberg, M. and Koch, S. W. Theory of coherent transients in semiconductor pump–probe spectroscopy. *JOSA B* **5**,139-146 (1988).

[3] Koch, S. W., N. Peyghambarian and M. Lindberg. Transient and steady-state optical nonlinearities in semiconductors. *Journal of Physics C: Solid State Physics* **21**, 5229 (1988).

[4] Sokoloff, J. P. et al. Transient oscillations in the vicinity of excitons and in the band of semiconductors. Physical Review B **38**, 7615 (1988).

[5] Hayat, Alex, et al. Dynamic Stark effect in strongly coupled microcavity exciton polaritons. *Physical Review Letters* **109**, 033605 (2012).

[6] Lange, Christoph, et al. Ultrafast control of strong light–matter coupling. *New Journal of Physics* **20,** 013032 (2018).

[7] Panna, Dmitry, et al. Ultrafast manipulation of a strongly coupled light–matter system by a giant AC Stark effect. *ACS Photonics* **6**, 3076-3081 (2019).

[8] Nathan, V. et al., Review of multiphoton absorption in crystalline solids. *Journal of the Optical Society of America B* **2**, 294-316 (1985).

[9] Bennett, B. R. et al., Carrier-induced change in refractive index of InP, GaAs and InGaAsP. *IEEE Journal of Quantum Electronics* **26**, 113-122 (1990).

[10] Tempel, J,S. et al. Characterization of two-threshold behavior of the emission from a GaAs microcavity. *Physical Review B - Condensed Matter and Materials Physics* **85**, 075318 (2012).
